# Casein as rare elicitor of food allergy dependent on augmentation factors ‐ A case report

**DOI:** 10.1111/ddg.70275

**Published:** 2026-03-22

**Authors:** Charlotte Jasmin Kiani, Valentina Faihs, Julia Felicitas Pilz, Claudia Kugler, Tilo Biedermann, Knut Brockow

**Affiliations:** ^1^ Department of Dermatology and Allergology Technical University of Munich Munich Germany; ^2^ Odense Research Center for Anaphylaxis (ORCA) Department of Dermatology and Allergy Centre Odense University Hospital Odense Denmark

**Keywords:** anaphylaxis, casein, FALDA, FDEIA, food allergy

Dear Editors,

A 31‐year‐old female patient with atopic dermatitis in childhood and mild allergic rhinoconjunctivitis to grass pollen presented at our clinic with a 10‐year history of recurrent systemic allergic reactions during physical exertion. Symptoms included tingling of extremities, generalized urticaria, throat swelling, abdominal pain, diarrhea, vomiting and in one case, loss of consciousness. In total, approximately 16 systemic episodes occurred, predominantly at warm temperatures. Between the reactions, physical exercise was tolerated. Initial suspicion of WALDA (wheat allergy dependent on augmentation factors) led to a strict wheat‐free diet for 1.5 years, without clinical improvement. The most recent two episodes occurred two months prior to presentation. In the first episode, she consumed a salad containing various vegetables, mozzarella, and sunflower seeds and went for a run two hours later. During the run, she developed generalized urticaria, angioedema, gastrointestinal symptoms and finally loss of consciousness, requiring antiallergic treatment by an emergency physician. Two weeks later, with a mild upper respiratory infection, she experienced a similar reaction during physical exercise two hours after consuming halloumi, fennel, parsley, and chickpeas.

Skin prick tests (SPT; prick‐to‐prick method) showed positive reactions to several cow´s milk products, including quark (3 mm wheal/5 mm erythema), mozzarella (3/10), a high‐protein milk drink (3/10), and yogurt (5/17) (Supplementary Figure S1). SPT to halloumi and to other foods consumed before the reactions (sunflower seed, chickpea, parsley, fennel) remained negative. Serology revealed normal total IgE levels (25 kU/L), but elevated specific IgE (sIgE; ImmunoCAP assay, Thermo Fisher Scientific, Uppsala, Sweden) to milk casein (nBos d 8; 0.61 kU/L). No sIgE were found for total milk protein, α‐lactalbumin, β‐lactoglobulin and for other foods consumed prior to reactions (e.g. chickpeas, different vegetables) as well as Pru p 3, alpha‐gal, and omega‐5‐gliadin (all < 0.1 kU/l).

According to current guidelines, oral food challenge tests (OFC) were performed (Figure 1).^1^ First, a combination of augmentation factors alone (1 g acetylsalicylic acid (ASA), 20 mL 95 % alcohol diluted in tea (Braun, Melsungen, Deutschland), 25 minutes intensive running on treadmill) was tolerated without any symptoms. Also, ingestion of 50 g halloumi (a pre‐cooked cheese containing heat‐stable casein), and subsequently 150 g combined with 1 g ASA, were both tolerated without symptoms. On the following day, ingestion of 105 g halloumi preceded by 1 g ASA and 20 mL of 95 % alcohol was followed by treadmill exercise. After 10 minutes of running, the patient developed intense pruritus, generalized urticaria, and eyelid angioedema. Shortly thereafter, she experienced nausea, globus sensation, dizziness, shivering, hypotension (89/52 mmHg; initial 128/61 mmHg) and tachycardia (139 bpm). Immediate antiallergic treatment with 300 µg intramuscular adrenaline and intravenous dimetindene maleate (8 mg) and prednisolone (250 mg) was initiated, which led to complete resolution of symptoms. A significant rise in serum tryptase (baseline 3.4 µg/L; 30 min after reaction: 12.6 µg/L; 90 min: 18.3 µg/L) confirmed anaphylaxis. Thus, the diagnosis of casein‐induced FALDA (food allergy dependent on augmentation factors) was confirmed. The patient received dietary counselling and was advised to avoid cow´s milk products in high amounts and in combination with augmentation factors.

**FIGURE 1:**
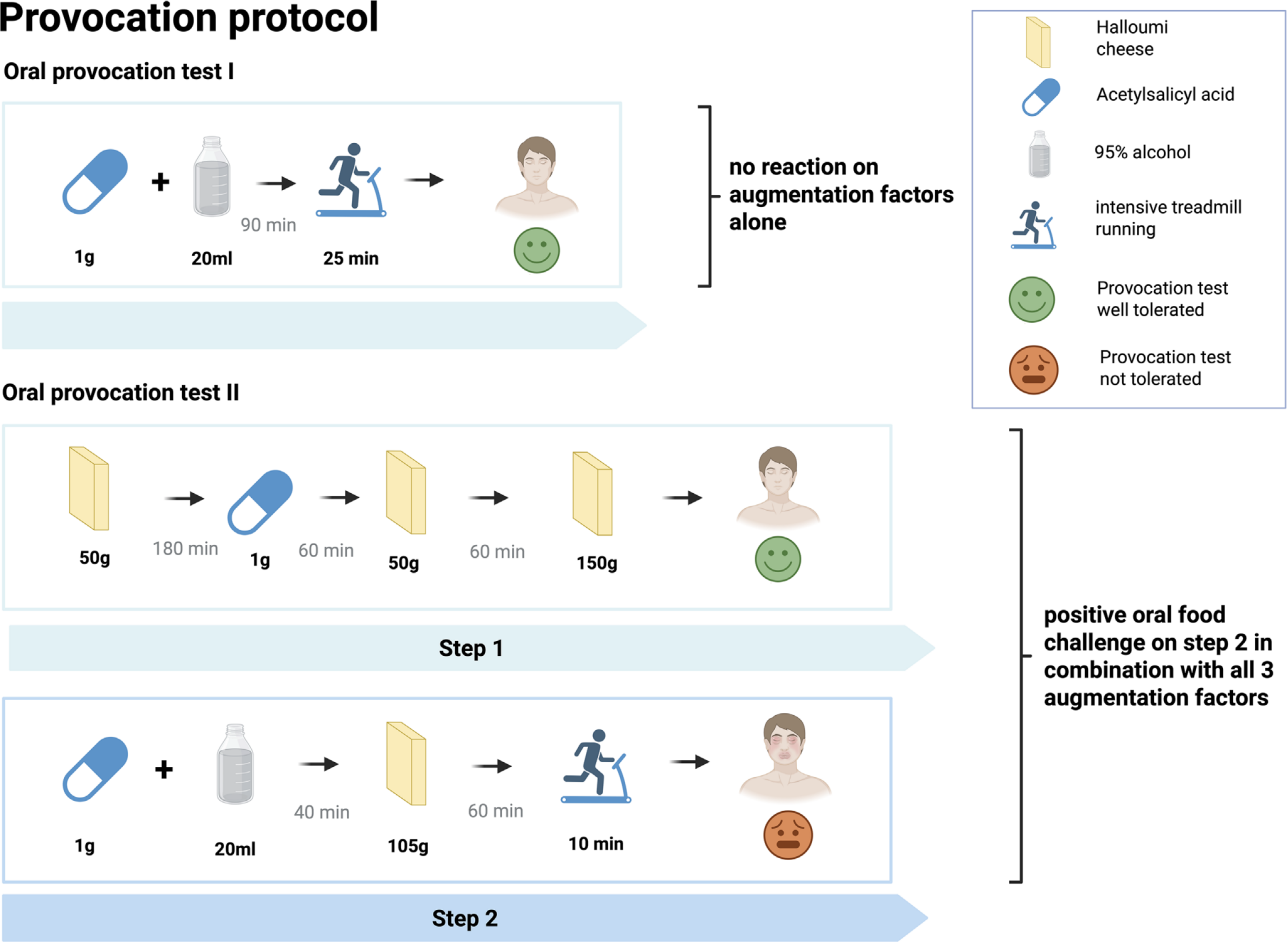
Provocation protocol of the oral challenge test. Oral provocation test I: All augmentation factors were tested without any food to exclude an exercise‐induced anaphylaxis. Oral provocation test II: Oral food challenge with halloumi to verify casein‐induced FDEIA. Created in BioRender. Köberle, M. (2025) https://BioRender.com/4ml4oag.

Food allergy dependent on augmentation factors (FALDA) is a specific form of IgE‐mediated food allergy occurring only when ingestion of the culprit food coincides with augmentation factors such as physical exercise, nonsteroidal anti‐inflammatory drugs or alcohol consumption.^2^, ^3^ While cow´s milk proteins, in particular casein and the whey proteins alpha‐lactalbumin and beta‐lactoglobulin are well‐known triggers in classic IgE‐mediated food allergy and not dependent on augmentation factors, their role in FALDA is rarely documented. Casein represents the predominant, heat stable fraction (80 %), whereas whey constitutes the remaining heat‐labile 20 % of protein in cow´s milk.^4^ All dairy products contain relevant amounts of casein, although in varying concentrations depending on processing. Among the products tested in this case, cheese generally contains the highest concentration of casein. However, extensive fermentation and maturation sometimes may reduce allergenicity due to structural modification.^5^ This could be one reason for the negative SPT to the tested halloumi and for the need of augmentation factors in conjunction with consumption of higher amounts of halloumi to trigger a clinical reaction.

The first case of cow´s milk‐induced FALDA was reported in 2003, describing a patient with primary IgE‐mediated cow´s milk allergy in childhood, which later evolved into FALDA during adolescence.^6^ This contrasts with the history of our patient, who did not suffer from cow´s milk allergy in her childhood and who first developed FALDA in adulthood. Adult‐onset food allergy is often described in FALDA, especially in the most frequent form of WALDA.^7^

Another published case of cow´s milk‐induced FALDA demonstrated positive OFC only during the ovulatory phase, underscoring the complexity of finding a correct diagnosis.^8^ In our patient, no dependency on the menstrual cycle was reported in her history. However, a single augmentation factor alone (1 g ASA) was not sufficient to elicit symptoms. OFC was positive only with a combination of different augmentation factors.

Notably, in our patient, casein‐specific IgE levels were only modestly elevated (0.61 kU/L; total IgE within the normal range (25 kU/L)) and SPT to halloumi was negative. Nevertheless, she reacted unequivocally and severely during OFC as well as in her clinical history. This shows that diagnosis and severity do not necessarily correlate with sensitization levels, and that in some individuals augmentation factors may fundamentally lower the reaction threshold.^2^, ^9^ OFC therefore remains essential for diagnosis, to avoid further, life‐threatening events and to help restore the patients´ quality of life by identification of the culprit trigger.^2^, ^3^, ^10^ Also in our patient, the diagnosis was delayed by almost ten years, leading to multiple severe reactions.

Taken together, this case illustrates a rare form of casein‐induced FALDA, confirmed by OFC. It highlights the need to consider casein as a potential trigger not only in classical cow´s milk allergy, but also in FALDA. Furthermore, it underlines the diagnostic challenges in FALDA, showing that low‐grade sensitization can become clinically relevant in the presence of augmentation factors and underscoring the need for OFC with augmentation factors for correct diagnosis.

## CONFLICT OF INTEREST STATEMENT

None.

## FUNDING

This work was supported by the German Federal Ministry of Research, Technology and Space (BMTR) as part of the ABROGATE project (grant number 01EA2106A) awarded to KB, by the Clinician Scientist Program TRIAL of the German Society for Allergy and Clinical Immunology (DGAKI) to VF as well as by the German Research Foundation (Deutsche Forschungsgemeinschaft, DFG) within the RTG 2668 framework (Project A3 and A4, Project‐ID: 435874434).

## Supporting information



Supplementary information

Supplementary information
