# Supplementary material for: Casein as rare elicitor of food allergy dependent on augmentation factors ‐ A case report
Source: J Dtsch Dermatol Ges. 2026 Mar 22;24(11):1650–2. doi: 10.1111/ddg.70275 (PMC13749601; doi:10.1111/ddg.70275)
Supplement: Supplementary file 1 [file DDG-24-1650-s001.pdf]

Skin prick test

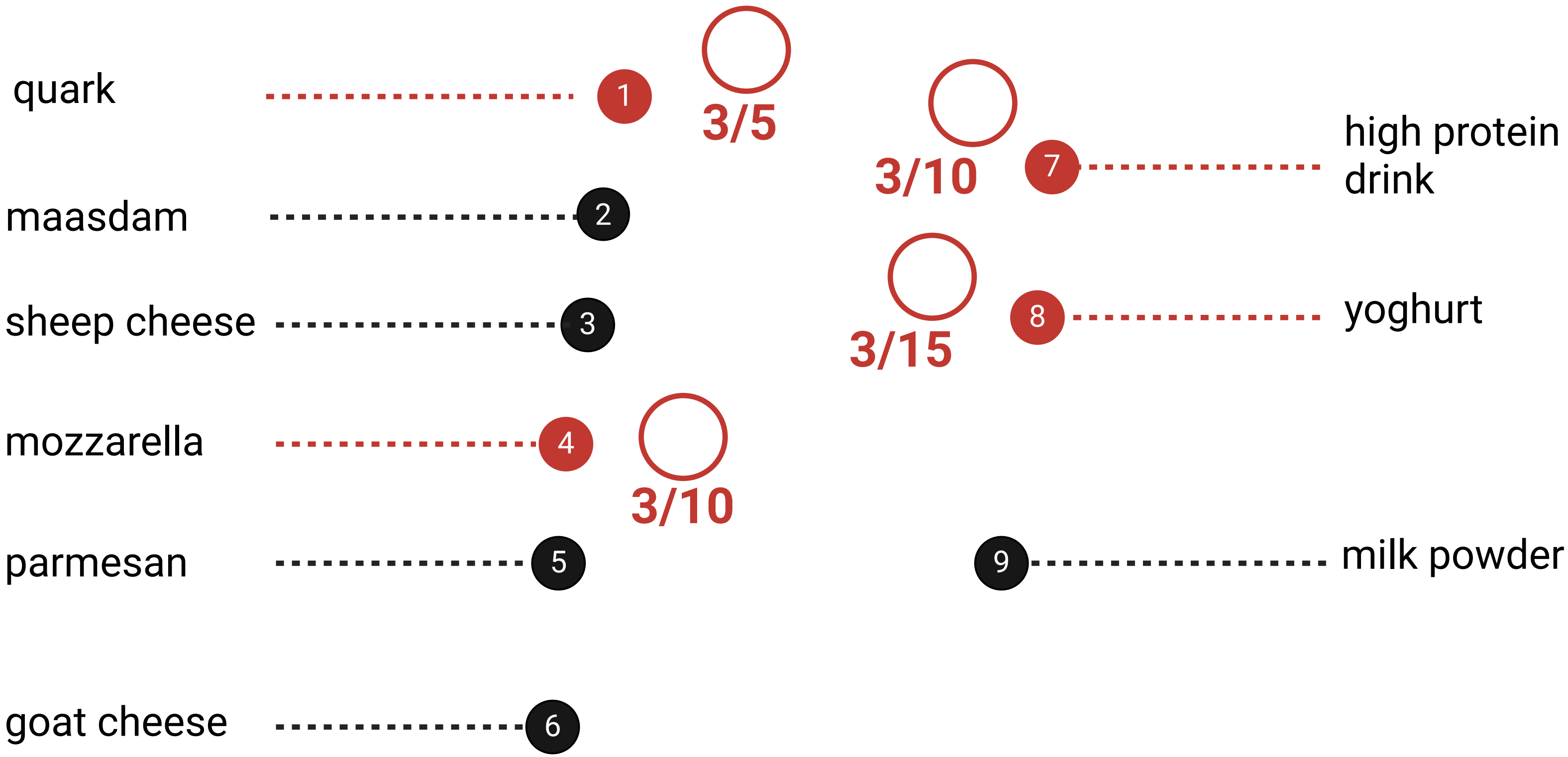

Legend prick test results:
mm wheal/mm erythema
